# Multi-index fuzzy comprehensive evaluation model with information entropy of alfalfa salt tolerance based on LiDAR data and hyperspectral image data

**DOI:** 10.3389/fpls.2023.1200501

**Published:** 2023-08-17

**Authors:** Jiaxin Zhang, Aiwu Zhang, Zixuan Liu, Wanting He, Shengyuan Yang

**Affiliations:** ^1^ Key Laboratory of 3D Information Acquisition and Application, Ministry of Education, Capital Normal University, Beijing, China; ^2^ Engineering Research Center of Spatial Information Technology, Ministry of Education, Capital Normal University, Beijing, China; ^3^ Center for Geographic Environment Research and Education, College of Resource Environment and Tourism, Capital Normal University, Beijing, China

**Keywords:** alfalfa, hyperspectral image, LiDAR data, phenotypic traits, the evaluation of salt tolerance, fuzzy

## Abstract

Rapid, non-destructive and automated salt tolerance evaluation is particularly important for screening salt-tolerant germplasm of alfalfa. Traditional evaluation of salt tolerance is mostly based on phenotypic traits obtained by some broken ways, which is time-consuming and difficult to meet the needs of large-scale breeding screening. Therefore, this paper proposed a non-contact and non-destructive multi-index fuzzy comprehensive evaluation model for evaluating the salt tolerance of alfalfa from Light Detection and Ranging data (LiDAR) and HyperSpectral Image data (HSI). Firstly, the structural traits related to growth status were extracted from the LiDAR data of alfalfa, and the spectral traits representing the physical and chemical characteristics were extracted from HSI data. In this paper, these phenotypic traits obtained automatically by computation were called Computing Phenotypic Traits (CPT). Subsequently, the multi-index fuzzy evaluation system of alfalfa salt tolerance was constructed by CPT, and according to the fuzzy mathematics theory, a multi-index Fuzzy Comprehensive Evaluation model with information Entropy of alfalfa salt tolerance (FCE-E) was proposed, which comprehensively evaluated the salt tolerance of alfalfa from the aspects of growth structure, physiology and biochemistry. Finally, comparative experiments showed that: (1) The multi-index FCE-E model based on the CPT was proposed in this paper, which could find more salt-sensitive information than the evaluation method based on the measured Typical Phenotypic Traits (TPT) such as fresh weight, dry weight, water content and chlorophyll. The two evaluation results had 66.67% consistent results, indicating that the multi-index FCE-E model integrates more information about alfalfa and more comprehensive evaluation. (2) On the basis of the CPT, the results of the multi-index FCE-E method were basically consistent with those of Principal Component Analysis (PCA), indicating that the multi-index FCE-E model could accurately evaluate the salt tolerance of alfalfa. Three highly salt-tolerant alfalfa varieties and two highly salt-susceptible alfalfa varieties were screened by the multi-index FCE-E method. The multi-index FCE-E method provides a new method for non-contact non-destructive evaluation of salt tolerance of alfalfa.

## 1 Introduction

Alfalfa is an important supplementary crop and plays an important role in animal husbandry. However, saline-alkali land has a serious impact on the growth and development of alfalfa (Munns and Tester, 2008). Therefore, it is of great practical significance to accurately evaluate the salt tolerance of alfalfa (Zhang et al., 2013; Reddy et al., 2022). However, it is a complex task to accurately evaluate the salt tolerance of alfalfa. Traditional phenotypic survey methods are limited by the number of samples and time costs, and cannot comprehensively evaluate the structural, physical and chemical indicators of alfalfa (Munns and Tester, 2008; Hanin et al., 2016; Ismail and Horie, 2017). Therefore, it is of great significance to comprehensively evaluate the salt tolerance of alfalfa by using modern plant phenotypic techniques to understand the salt tolerance mechanism of alfalfa, improve alfalfa varieties and increase agricultural production in saline-alkali areas (Hu and Schmidhalter, 2023).

Phenotypic traits of plant salt tolerance refer to all traits that reflect the physical, physiological and biochemical properties of plant salt tolerance influenced by genes or environment. Studies have been carried out in China and abroad to evaluate the salt tolerance of alfalfa using the phenotypic traits of indicator plant individuals or populations (Houle et al., 2010; Chunjiang, 2019; Al-Tamimi et al., 2022; Singh and Bhutia, 2022). However, considering the wide planting range of alfalfa, there are many drawbacks in the phenotype based on manual measurement. For example, large-scale monitoring will make the measurement cycle long and the timeliness of data low; point monitoring will make the data lack of overall representativeness, and it is difficult to meet the needs of regional breeding work. In addition, in breeding experiments, laboratory chemical analysis is often used to obtain physiological and biochemical parameters of alfalfa, which greatly increases the experimental cost. In order to promote the study of modern plant phenotypes, this paper proposes the concept of computing phenotypic traits. Computing phenotypic traits refer to the digital phenotypic traits that characterize the growth and development, physiology and biochemistry of plant individuals or populations extracted by comprehensive computer technology, image analysis technology and other modern science and technology. With the development of non-contact information collection methods such as computer vision, HSI technology, and 3D laser technology, more and more plant CPT have been extracted, which provides technical support for real-time and non-destructive evaluation of alfalfa salt tolerance (Tmušić et al., 2020; Brook et al., 2021; Singh et al., 2021; Li et al., 2022).

When alfalfa is subjected to salt stress, it first affects the physical and chemical parameters. The spectral absorption and reflection characteristics of plants can be used to characterize their physiological and biochemical characteristics (Tucker, 1977). Spectral indices can highlight phenotypic traits such as plant biomass, leaf water content, pigment content and salt stress index through band combination, and reduce the negative impact of spectral redundancy on trait extraction (El-Hendawy et al., 2022). The RGB and multispectral sensors have lower spectral resolution, fewer bands, and discontinuous spectral coverage, which results in a limited availability of spectral index features. HSI can describe the interaction between alfalfa physicochemical traits and the environment in more detail through its fine spectral superiority and spatial information, and shows a strong advantage in the extraction of alfalfa computational phenotypic traits (El-Hendawy et al., 2019a; Xiaofeng et al., 2020; Jin et al., 2021b). The hyperspectral narrow band vegetation index can more comprehensively characterize the content of crop stress resistance components through band combination (Post et al., 2007; Wu et al., 2008; Ullah et al., 2012; Thenkabail et al., 2013; El-Hendawy et al., 2019b), which provides an effective means for the study of alfalfa stress resistance phenotypic traits, breeding screening and implementation of precision agriculture (Hunt et al., 2013; Kasim et al., 2017). With the cumulative change of physical and chemical parameters, the growth, development and structural parameters of alfalfa were also affected and changed (Hanin et al., 2016). LiDAR technology measures the distance between the sensor and the target object by laser irradiation and is widely used in plant reconstruction and morphological structure extraction (M et al., 2021; Shaochen et al., 2023). Sun et al. used LiDAR technology to monitor the maximum canopy height, projected canopy area and plant volume of cotton plants with good results (Shangpeng et al., 2018). With the advancement of technology, the use of LiDAR point cloud data to obtain plant structural phenotypic traits has been widely recognized (Jimenez-Berni et al., 2018; Jin et al., 2021a). Secondly, LiDAR is an active remote sensing technology. Compared with spectral imaging technology, it is not affected by light and shooting angle, and can realize all-day monitoring. The true morphology of alfalfa was restored to the greatest extent by a high-precision three-dimensional point cloud, so as to extract its structural phenotypic traits (Shaochen et al., 2023). These structural phenotypic traits are essential for evaluating the salt tolerance of alfalfa. In this paper, plant height, canopy leaf area and volume extracted from LiDAR data, and special indicators extracted from HSI data were called computing phenotypic traits.

The salt tolerance response mechanism of alfalfa is complex. The evaluation of salt tolerance is based on the overall performance of various physiological processes, and any single trait can’t directly represent its salt tolerance level. It is more scientific and reasonable to use a multi-index system to comprehensively evaluate alfalfa (Shaohua et al., 2022; Sun et al., 2022). Some scholars have proposed that the method of a comprehensive evaluation of multiple indicators, such as cluster analysis, principal component analysis and the membership function value method, is the best method for screening salt-tolerant alfalfa (Hu et al., 2018; Xiangfeng et al., 2018). However, these evaluation methods are easily affected by subjective factors. Fuzzy Comprehension Evaluation (FCE) is a method based on the fuzzy transformation principle in fuzzy mathematics (Zadeh., 1965). Based on the principle of membership, the algorithm scientifically and objectively synthesizes a multi-index problem into a single-index result containing multi-index information, to realize multi-factor comprehensive evaluation in one-dimensional space (Robati and Rezaei, 2021). The basis of constructing a comprehensive evaluation model of salt tolerance by the FCE method is to establish a scientific, reasonable, comprehensive and objective evaluation index system. Xu et al. proposed a comprehensive yield evaluation index that reflected leaf area index, leaf biomass, leaf moisture content and leaf nitrogen content (Xu et al., 2021). However, the measurement of these indicators has low efficiency, serious subjectivity, large measurement error, and poor plant adaptability when repeated measurement of a single plant, which make it difficult to meet the requirements of modern agricultural production practice. In addition, these indicators focus on the estimation of yield and cannot represent the salt tolerance of alfalfa growth. Wu et al. discussed the contribution of morphological structure, physiological and biochemical phenotypic trait indicators to the screening of alfalfa germplasm. The results showed that the structural phenotypic traits associated with alfalfa growth and development and the phenotypic traits characterized by physicochemical characteristics were important parameters for evaluating the salt tolerance of alfalfa (Duan et al., 2008; Tilly et al., 2013; Tilly et al., 2015; Xinming et al., 2018; Duo et al., 2021; Penglei et al., 2021; Guiza et al., 2022). However, phenotypic traits based on manual measurement and chemical analysis are expensive and limited. Therefore, the application of remote sensing technology to the evaluation of salt tolerance can not only extract more phenotypic traits to characterize alfalfa salt tolerance more efficiently but also avoid irreversible damage to alfalfa caused by measurement in indicators.

Another focus of the FCE method is the weight distribution of the evaluation indicators. The traditional method of determining the weight mostly adopts the expert scoring method, which has strong subjective defects (Du et al., 2019). Therefore, it is necessary to improve the FCE method to more objectively reflect the salt tolerance level of alfalfa. In summary, the screening of salt-tolerant varieties is based on the comprehensive evaluation of their phenotypic traits. However, most of the current research on the salt tolerance evaluation of alfalfa relies on TPT (Roy et al., 2014; Ismail and Horie, 2017), and few studies have applied remote sensing technology to the screening of alfalfa salt tolerant varieties. Moreover, the construction of the comprehensive evaluation model of salt tolerance is mostly affected by subjective factors (Song et al., 2021). Therefore, the purpose of this study is to conduct a rapid and non-destructive comprehensive evaluation of alfalfa salt tolerance at flowering date based on HSI and LiDAR data, and to improve the FCE method to enhance the automation ability of the salt tolerance evaluation model. The specific research objectives of this study are as follows: (1) To construct a digital and non-destructive evaluation index system of alfalfa salt tolerance that characterizes the growth, development and physicochemical characteristics of alfalfa; (2) It is proven that the CPT can capture more salt sensitive information than the TPT and is more comprehensive and robust in evaluating the salt tolerance of alfalfa. (3) The FCE method was optimized to develop an automated comprehensive evaluation model for salt tolerance of alfalfa with strong portability. (4) Compared with the PCA method, the multi-index FCE-E model is more suitable for screening and breeding research. This study not only helps to enhance the understanding of the salt tolerance mechanism of alfalfa, but also provides new ideas and approaches for the evaluation of the salt tolerance of alfalfa, and provides valuable references for research and practice in related fields.

## 2 Materials and methods

### 2.1 Design of experiment

In this experiment, six alfalfa varieties of WL343HQ, Gibraltar, Gold Empress, Zhongmu No. 3, Aohan, and Cangzhou were studied at flowering date. To compare the effects of salt stress on the growth status of alfalfa, a salt stress (NaCl) group and a control (CK) group were set up on the material, and a repeat experiment was set up at the same time. In the pre-experiment, it was found that the differences between alfalfa varieties were not prominent in the salt stress treatment of 100 mmol/L; with 150 mmol/L of salt stress, there were obvious differences between alfalfa varieties. All varieties survived in two pre-experiments. Therefore, the NaCl group in this study was treated with salt stress of 125 mmol/L, and the CK group was treated with clean water.

**Figure 1:**
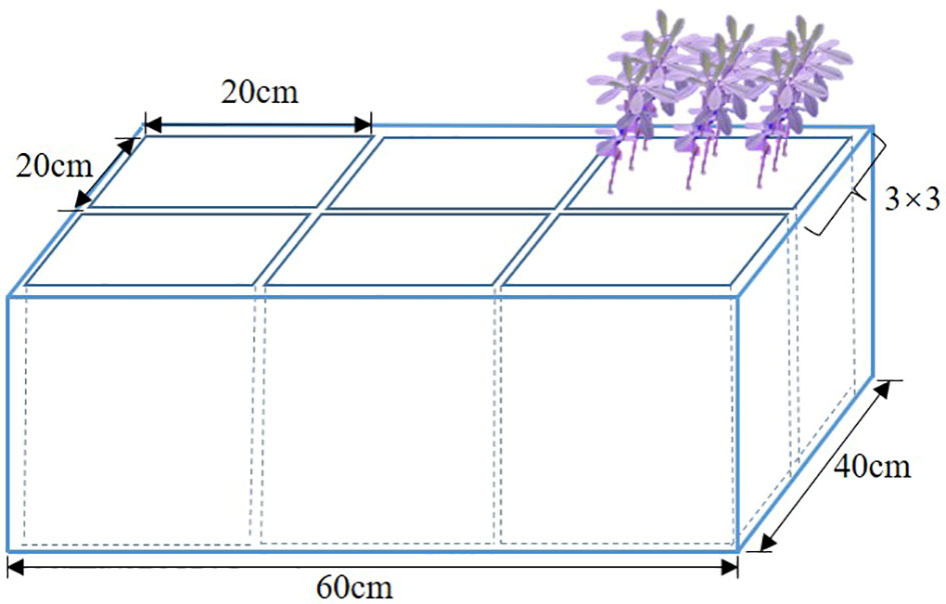
Schematic diagram of alfalfa planting.

To avoid the effect and damage caused by watering impulse on alfalfa roots, 9 plants of the same variety of alfalfa were evenly planted uniformly in a 20 cm × 20 cm porous pot, and 6 porous pots were placed in a 40 cm × 60 cm non-porous box (**Figure 1**). The irrigation treatment was in the non-porous box. Porous pots could enable alfalfa connections to be placed in a habitat, reducing the generation of variables. Except for different treatment methods, the other growth environments of the sample materials were consistent, which met the experimental conditions of the single variable method.

To construct a comprehensive evaluation model of salt tolerance based on non-destructive monitoring, before measuring the measured phenotypic traits such as fresh weight and dry weight of alfalfa at flowering date, HSI data and LiDAR data were collected, and the spectral and structural phenotypic traits were analyzed and extracted.

### 2.2 Methods of research

**Figure 2:**
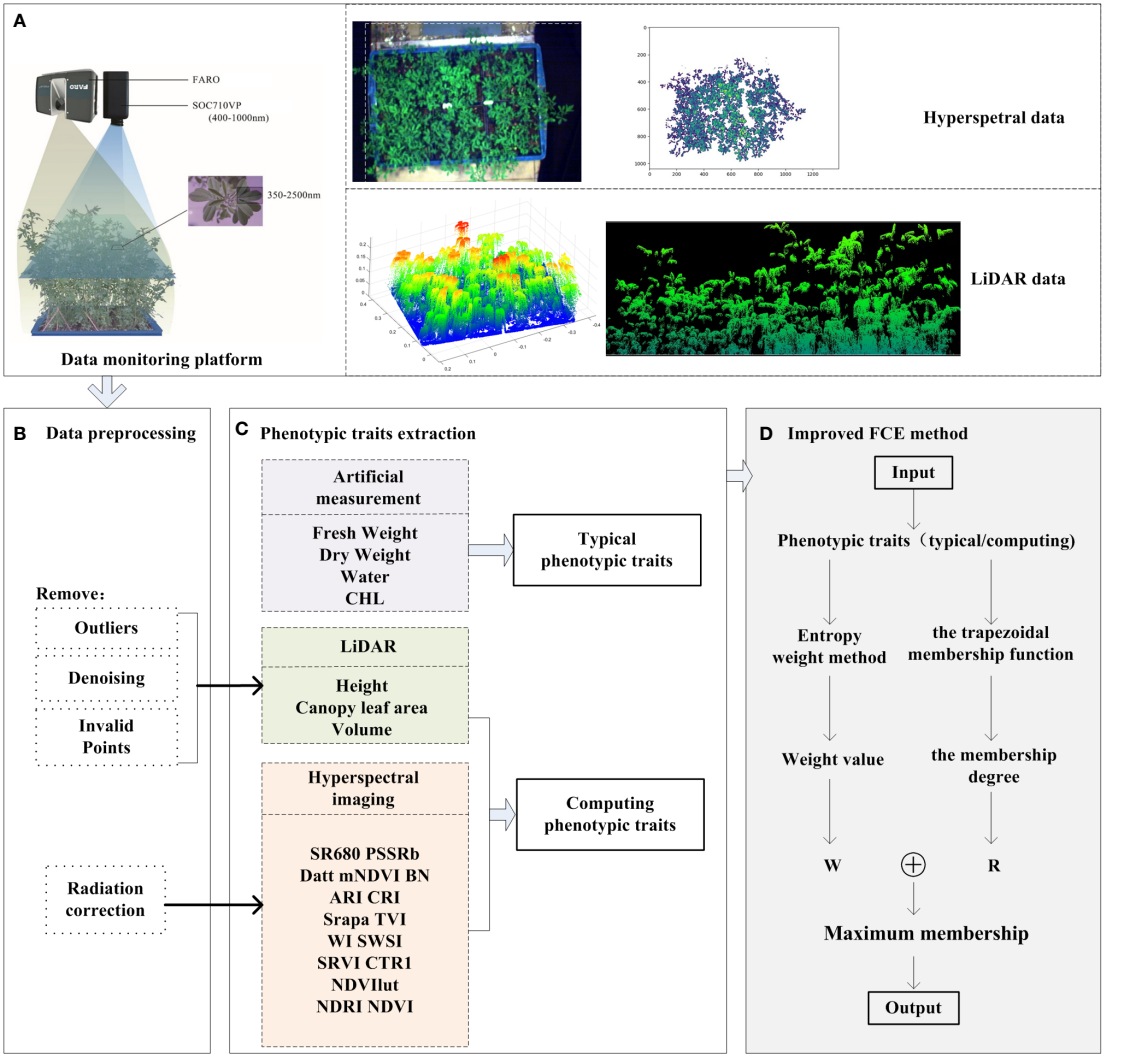
A workflow diagram of the experimental design, feature extraction, and modeling. **(A)** data collection; **(B)** data preprocessing for original data; **(C)** phenotypic traits extraction; **(D)** improved FCE method and model construction.

**Figure 3:**
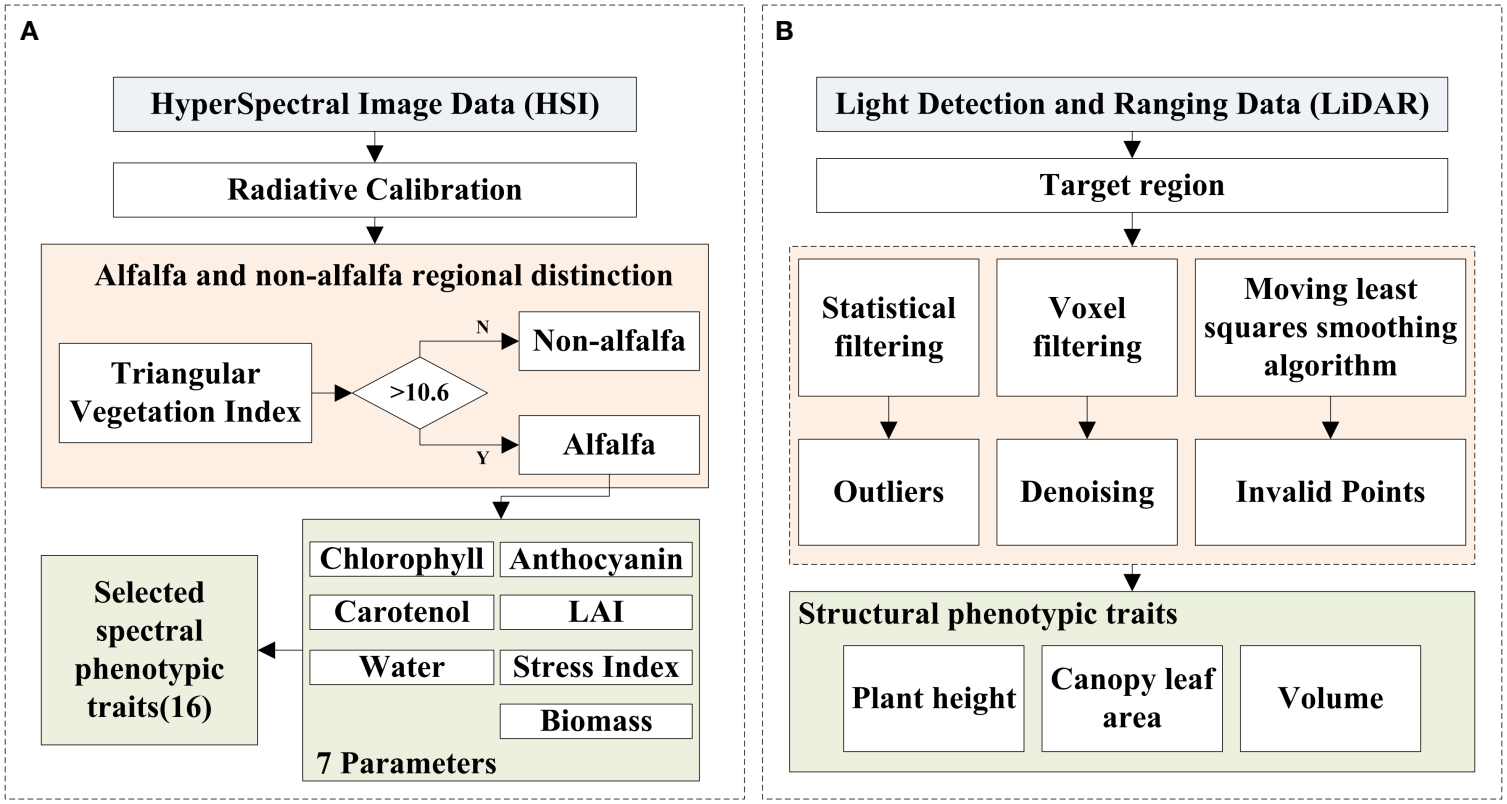
The processing flow chart of HSI data and LiDAR data. **(A)** is the HSI data, **(B)** is the LiDAR data.

To construct a salt tolerance evaluation model of alfalfa and screen the salt-tolerance alfalfa germplasm resources, it is necessary to establish a multi-index salt tolerance evaluation system for alfalfa. The experimental process is shown in **Figure 2**, which mainly consists of four main parts: (1) Data collection: To achieve rapid and non-destructive evaluation of alfalfa’s salt tolerance, HSI data and LiDAR data of alfalfa at flowering date were collected. In addition, four typical traits of alfalfa, including Fresh Weight (FW), Dry Weight (DW), Water Content (WC) and Chlorophyll (SPAD, CHL), were collected after mowing. (2) Data preprocessing for original data: In order to improve the accuracy of extracting structural phenotypic traits from the LiDAR data, the outliers, denoising and invalid points were removed from the LiDAR data, and then the structural phenotypic traits of alfalfa were extracted; The radiometric calibration of the HSI data was carried out by using the software of the hyperspectral imager, and then the spectral phenotypic traits of alfalfa were extracted (**Figure 3**). (3) Phenotypic traits extraction: Construct the CPT using the structural phenotypic traits extracted from the LiDAR data and the spectral phenotypic traits extracted from the HSI data. (4) Improved FCE method and model construction: Firstly, the subjectivity of the FCE method is improved by the entropy weight method and adaptive adjustment of the critical point of the fuzzy membership function. Then, on the basis of the CPT (step 3), comprehensive evaluation models for salt tolerance of alfalfa in the CK group and NaCl group were constructed by the multi-index FCE-E method, to obtain the salt tolerance rating of alfalfa and screen out the salt tolerance germplasm resources.

#### 2.2.1 Acquisition of measured data

This experiment was based on the “Descriptors and Data Standard for Medic (Medicago Linn.) (Hongyan and Zongli, 2007)” investigated and extracted four typical phenotypic traits of alfalfa at flowering date, including FW, DW, WC, and CHL. First, the material was cut from 1cm above the ground in a non-porous box, and the alfalfa samples in each box were loaded into a mesh bag of the same specification. The FW was obtained by directly weighing each box of alfalfa plants and mesh bags using an electronic scale (± 0.01g). The DW was obtained by weighing the plant and drying it in a mesh bag at 120°C to a constant weight. The WC was the difference between FW and DW. The CHL was the average value of more than 15 measurements by the SPAD instrument.

#### 2.2.2 Acquisition and preprocessing of hyperspectral data

In this study, the SOC710VP hyperspectral imager with a spectral range of 400-1000 nm was used to collect hyperspectral data with a spectral resolution of 4.6875 nm. HSI data were acquired by setting up a darkroom and illuminating it with a full-band lamp. At the same time of data collection, a standard gray plate as high as alfalfa was placed next to alfalfa for radiometric calibration of HSI data.

The HSI preprocessing process is shown in **Figure 3A**. The original image has radiation errors, so radiation calibration was performed before use. Because the HSI data was collected indoors, the influence of the atmosphere on the calibration did not need to be considered. The relationship between the calibration data of the hyperspectral imager and the actual measured radiance brightness of each band is established by the software of the instrument to achieve the purpose of radiation correction.

**Figure 4:**
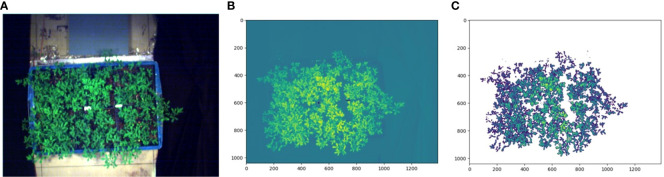
TVI Index distinguishing alfalfa and non-alfalfa. **(A)** is the RGB image synthesized by hyperspectral data, **(B)** is the TVI index image, **(C)** takes TVI = 10.6 as the threshold, the area with TVI greater than 10.6 is the alfalfa, and the area with TVI less than 10.6 is the other.

In order to extract the spectral phenotypic traits characterizing the physical and chemical characteristics of alfalfa, this paper first distinguishes alfalfa and non-alfalfa regions based on HSI data, and then the average value of the alfalfa imaging range was taken as the value of this trait. The experimental results have shown that the Triangular Vegetation Index (TVI, **Table 1**) could better distinguish between alfalfa and non-alfalfa in hyperspectral images (**Figure 4**). TVI is closely related to chlorophyll content. It is constructed according to the difference in light radiation energy of pigments in green, red and near-infrared radiation energy. A robust “triangle” spectral space is formed by the reflection peak of green light, the absorption valley near red light, and the red edge of the near-infrared. Vegetation and non-vegetation regions can be distinguished according to this spectral signature.

**Table 1:** Selected spectral traits.

Parameters	VI	Formular	Reference
Chlorophyll	Simple Ratio Index	SR680=R800/R680	(Sims and Gamon, 2002)
	Pigment-specific Simple Ratio	PSSRb=R800/R635	(Blackburn, 2010)
	Datt Index	Datt=(R850-R710)/(R850-R680)	(Datt, 2010)
	Modified Normalized Difference Vegetation Index	mNDVI=(R750-R705)/(R750+R705-2*R445)	(Sims and Gamon, 2002)
	BN Index	BN=Log(R800/R550)	(Buschmann and Nagel, 2007)
Anthocyanin	Anth Reflectance Index	ARI=1/R550-1/R700	(Gitelson et al., 2001)
Carotenol	Carotenoid Reflectance Index	CRI=(1/R510)-(1/R550)	(Gitelson et al., 2002)
LAI	Simple Ratio Index	Srapa=R900/R680	(Aparicio et al., 2002)
	Triangular Vegetation Index	TVI=0.5*(120*(R750-R550)-200*(R670-R550))	(Broge and Leblanc, 2001)
Water	Water Index	WI=R900/R970	(Penuelas et al., 2010)
	Salinity and Water Stress Indices	SWSI=(R803-R681)/((R905+R972)**0.5)	(Hamzeh et al., 2013)
Stress Index	Simple Ratio Vegetation Index	SRVI=R830/R660	(Wang et al., 2010)
	Carter Indices	CTR=R695/R420	(Carter, 2007)
	Normalized Difference Vegetation Index	NDVIlut=(R913-R711)/(R913+R711)	(Luther and Carroll, 1999)
	Normalized Difference Red Edge Index	NDRI=(R790-R720)/(R790+R720)	(Barnes et al., 2000)
Biomass	Normalized Difference Vegetation Index	NDVI=(R800-R670)/(R800+R670)	(Tucker, 1979)

Where R * is the reflectivity of the corresponding wavelength.

In the experiment, it was found that in the TVI image, the TVI values of alfalfa and non-alfalfa areas were quite different, while the TVI values in the alfalfa area were similar. The threshold segmentation method was the simplest and the most appropriate method for extracting alfalfa. Through the experimental method, 10.6 was set as the threshold of TVI to distinguish alfalfa and non-alfalfa areas. After alfalfa extraction, the average value of alfalfa imaging range after band calculation was used as the value of spectral traits to provide parameters for the multi-index salt tolerance evaluation model of this study.

#### 2.2.3 Acquisition and preprocessing of LiDAR data

In this study, a FARO Focus M70 laser scanner with a wavelength of 1550nm was used to obtain LiDAR point cloud data, and the point cloud was preprocessed according to the process shown in **Figure 3B**. LiDAR uses scanning to obtain three-dimensional information such as the structure, position and shape of the target object, and inevitably produces some noise. In this experiment, the target object was cut first, and then the point cloud data of the target object was preprocessed by removing outliers, redundant points and mixed points. First of all, when the LiDAR scanned and recorded the information of the measured object, random noise would be generated, or some objects were not completely scanned. In order to make the point cloud structure characteristics of the alfalfa group clearer, this paper used the statistical filtering of the standard deviation multiple to eliminate outliers. Secondly, the LiDAR scanned the same area repeatedly during the scanning process, resulting in redundant point clouds, which require high data storage space and hardware equipment. According to the growth characteristics of the alfalfa population, this paper performed voxel filtering on the point cloud below the canopy, which reduced the number of point clouds on the basis of ensuring the geometric characteristics of point clouds, saved storage space and improved the efficiency of parameter extraction. Finally, in order to highlight the spatial characteristics of the alfalfa point cloud and ensure the smooth surface of leaves, aiming at the characteristics of high overlap, small leaves, and serious inter-species interleaving in the alfalfa population, this paper used the moving least squares smoothing algorithm to fit the surface by setting the radius range and projecting the mixed points to the surface to remove the mixed points. The above preprocessing work enabled the point cloud data to more accurately reflect the true information of alfalfa and provided a high-quality data basis for the subsequent extraction of structural phenotypic traits.

### 2.3 The construction of the multi-index fuzzy evaluation system

The effects of salt stress on alfalfa are mainly manifested in two aspects: (1) effects on the structural growth and development of alfalfa; and (2) effects on physiological and biochemical characteristics of alfalfa. Therefore, a multi-index fuzzy evaluation system was constructed by CPT combining spectral phenotypic traits based on HSI data and structural phenotypic traits based on LiDAR data.

**Figure 5:**
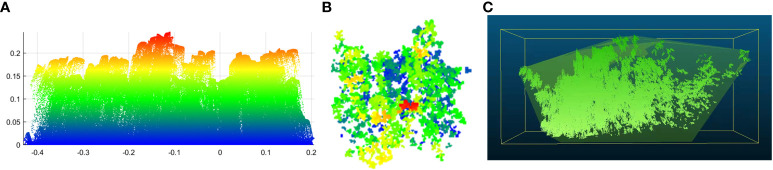
Structural phenotypic traits diagram. **(A)** is the plant height, **(B)** is the canopy leaf area, **(C)** is the volume.

The structural phenotypic traits related to the growth and development of alfalfa were extracted with three typical indexes: plant height, canopy leaf area and volume (**Figure 3B**). The change in plant height can reflect the change in alfalfa stem length, which is an important indicator of alfalfa growth; The change in canopy leaf area can reflect the speed of leaf growth. Alfalfa leaves provide nutrients for alfalfa growth through photosynthesis, which is very important for the growth and development, chlorophyll content and health status of alfalfa; Volume is the space occupancy of alfalfa growth, which is closely related to alfalfa biomass. In this paper, the bottom of alfalfa was used as the benchmark of point cloud data. Alfalfa was divided into 0.5cm grids, and the highest points in all grids were counted, and the average value was taken as the average plant height of alfalfa (**Figure 5A**). The sum of all grid areas was used as the canopy leaf area of the alfalfa population (**Figure 5B**). Alfalfa volume was calculated by constructing a convex hull (**Figure 5C**).

**Figure 6:**
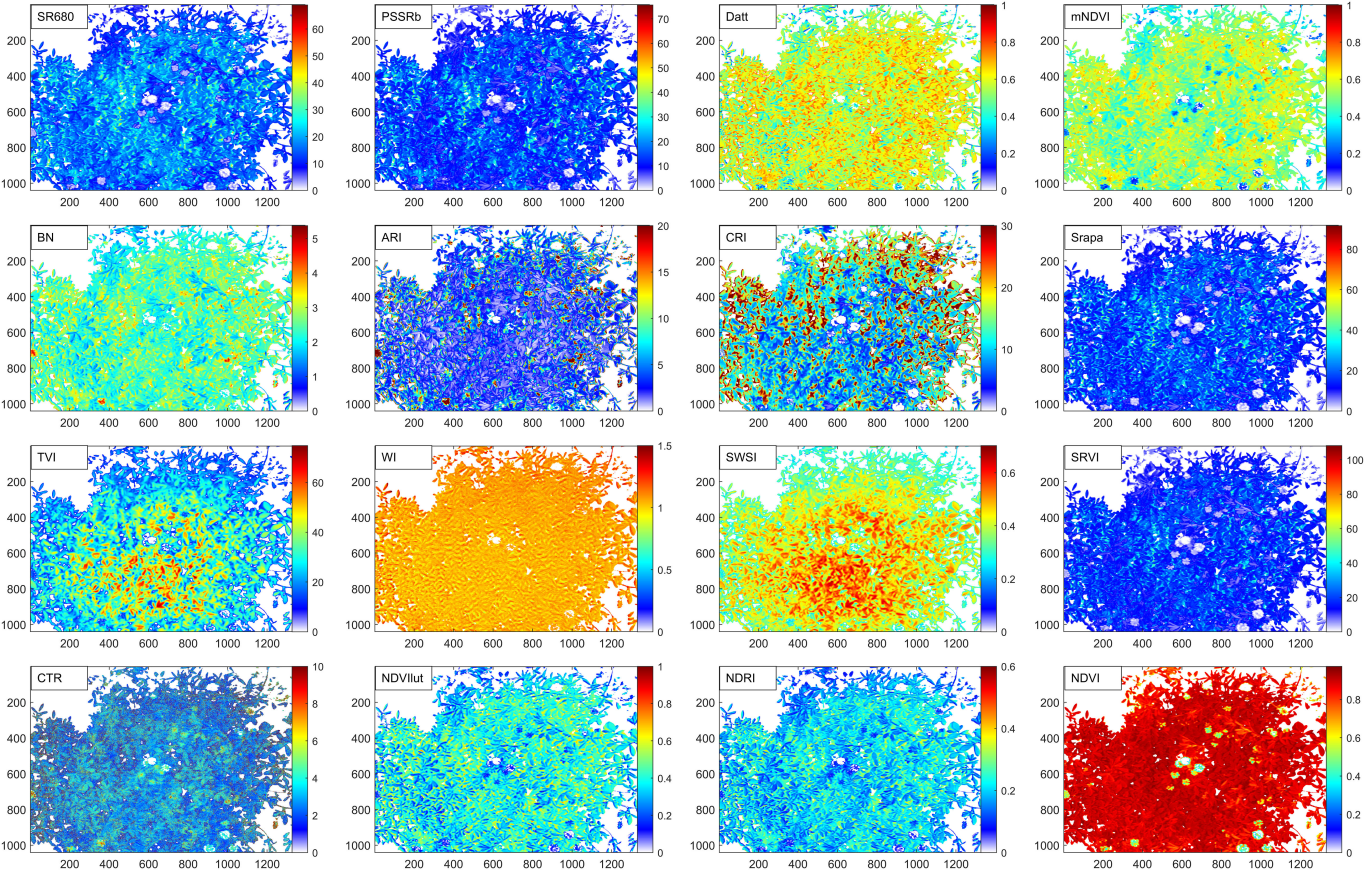
Spectral phenotypic traits diagram.

The spectral phenotypic traits were extracted by analyzing the spectral curve principle to characterize the phenotypic traits of alfalfa chlorophyll, anthocyanin, carotenol, leaf area index, water content, stress index and biomass (**Figure 3A**). Chlorophyll is involved in the absorption, transmission and transformation of light energy, which is an important basis for the growth of alfalfa. Water content is an important index in the evaluation of salt tolerance. Excessive salt accumulation in the soil will cause a decrease in soil water potential, making it difficult for alfalfa to absorb water, resulting in physiological drought and osmotic stress. The salt stress index is a comprehensive response to salt stress. Studies have shown that hyperspectral data can well reflect plant physiology and biochemistry information through a variety of linear or non-linear combination band operations (Saric et al., 2022). From the existing studies, a total of 16 vegetation indices were screened in this paper (**Table 1**, **Figure 6**), Among them, 5 indexes were extracted to characterize chlorophyll, 1 index was extracted to characterize anthocyanin, carotenol and biomass, 2 indexes were extracted to characterize leaf area index and leaf water content, and 4 indexes were extracted to characterize stress index. The vegetation index was calculated using the Python language GDAL library, and the alfalfa area extracted in the Section 2.2.2 was used as a mask, and the mean values of the alfalfa area of the vegetation indexes was used as the spectral phenotypic traits of this study.

### 2.4 The construction of fuzzy comprehensive evaluation model

**Figure 7:**
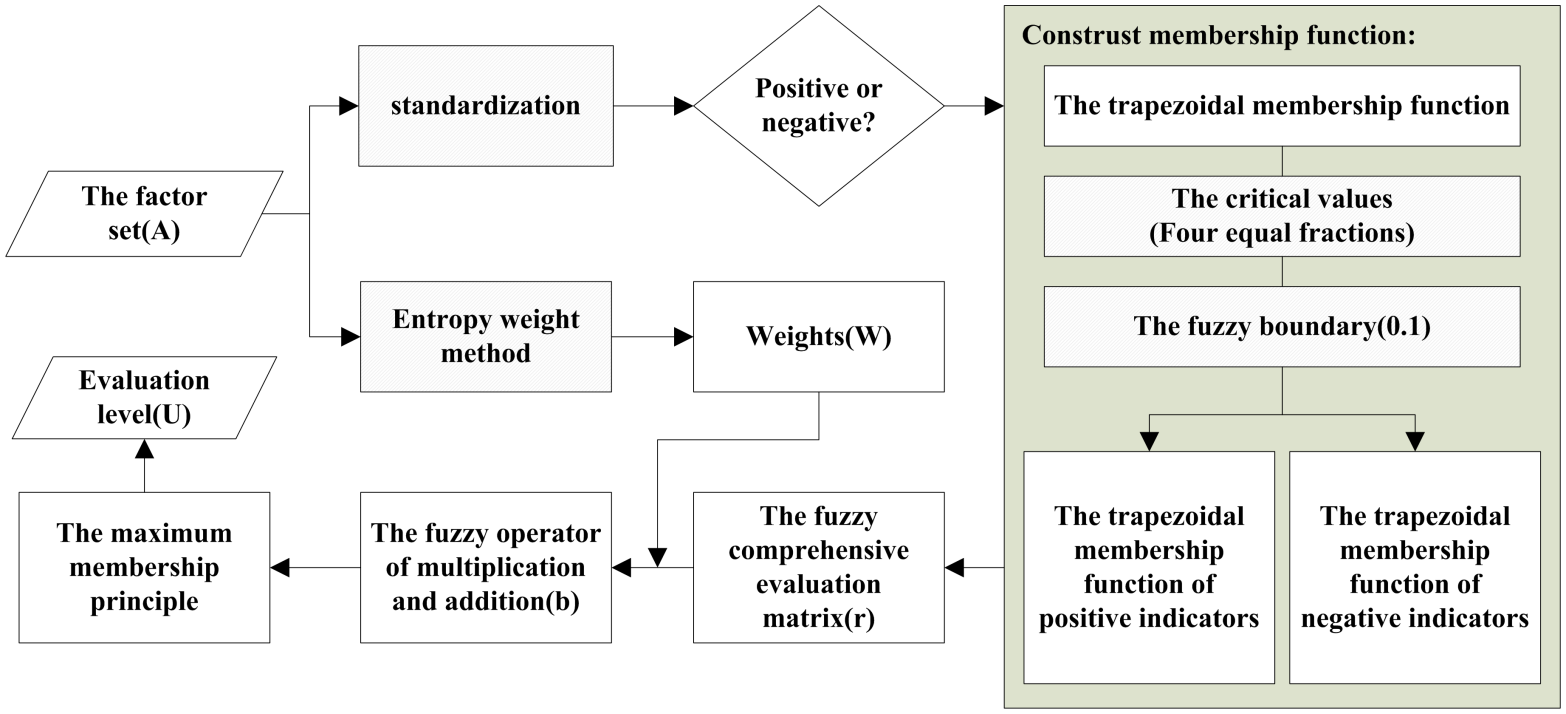
The construction of fuzzy comprehensive evaluation model.

Based on the multi-index fuzzy evaluation system (in section 2.3), the evaluation model of alfalfa salt tolerance was constructed by FCE-E method. This algorithm was completed on Matlab (**Figure 7**). The algorithm steps are as follows:

1. Input of the algorithm;

Let the evaluation index system be $\text{X}=\left[\begin{array}{cccc} X_{1} & X_{2} & \cdots & X_{m} \end{array}\right]$ , where *X_m_* is the mth evaluation index. In this paper, the fuzzy evaluation index system for salt tolerance (in section 2.3) was used as the factor set, and the data from the factor set was used to construct a two-dimensional matrix *A*, where *X_nm_* was the value of the mth index of the nth group of alfalfa.


(1) $$A=\left[\begin{array}{cccc} X_{11} & X_{12} & \cdots & X_{1\text{m}}\\ X_{21} & X_{22} & \cdots & X_{2\text{m}}\\ \vdots & \vdots & \ddots & \vdots \\ X_{n1} & X_{n2} & \cdots & X_{n\text{m}} \end{array}\right]$$

Let the evaluation set be $\text{U}=\left[u_{1}\;u_{2}\;\cdots \;u_{n}\right]$ , where *u_n_* is the nth evaluation level. In this paper, the evaluation set was set $\text{U}=\left[\begin{array}{cccc} Very\;tolerant & \;Intermidiate & \;Susceptible & \;Very\;susceptible \end{array}\right]$ according to the “Descriptors and Data Standard for Medic(Medicago Linn.) (Hongyan and Zongli, 2007)”.

2. Decide whether the evaluation index is positive or negative;

With yield as the evaluation reference, the evaluation index was positively correlated with yield, and the index was defined as a positive index. On the contrary, the evaluation index was negatively correlated with the yield, so the evaluation index was defined as a negative index.

3. The entropy weight method is used to calculate the weight distribution of the evaluation index system;

Due to the different contributions of each evaluation index to salt tolerance evaluation, different weights should be assigned to each factor involved in the evaluation. The entropy weight method is an objective weight method that determines the index weight according to the variation degree of each index value.

First, the index data was standardized and converted into a range between 0-1. The evaluation of salt tolerance focused on the difference in phenotypic traits among different alfalfa varieties under salt stress, and the weight of indexes was determined according to the different information contained in phenotypic traits. Therefore, the information entropy *E* (*X*) was calculated to measure the amount of information, and then the weight *W* (*X*) was determined according to the information entropy of each index, and finally the weight matrix *W*of the evaluation index system was constructed.


(2) $$E\left(X\right)=-\Sigma \left(P\left(X_{i}\right)*\log_{2}\left(P\left(X_{i}\right)\right)\right)$$

(3) $$W\left(X\right)=\frac{1-E\left(X\right)}{\Sigma \left(1-E\left(X\right)\right)}$$

(4) $$\text{W}=\left[\begin{array}{cccc} w_{1} & w_{2} & \ldots & w_{\text{m}} \end{array}\right]$$

Where, *X* is the index, *X_i_* is the value of the index *X* of each sample, and *P*(*X_i_*) is the probability of each value of the index *X_i_*. *W*(*X*) is the weight of the index *X*.

**Figure 8:**
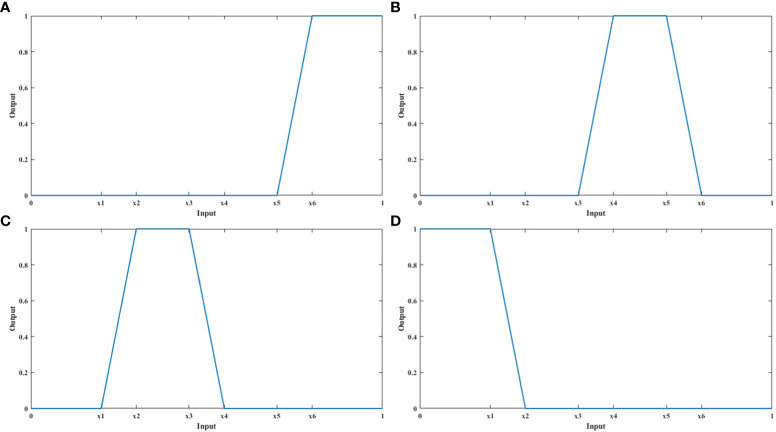
The fuzzy membership functions of the positive indicators were presented. **(A–D)** shows the fuzzy membership functions of four salt tolerance ratings: ‘Very tolerant’, ‘Intermediate’, ‘Susceptible’ and ‘Very susceptible’, respectively.

4. Construct membership function;

Each evaluation index presents different rules for the evaluation level. This study used the most commonly used trapezoidal membership function to set membership rules, and the parameters of membership function are set by adaptive parameter adjustment method. Firstly, the positive and negative indicators were uniformly standardized to the interval [0,1], to avoid the influence of outliers and extreme values indirectly through centralization, and to be basis of adaptive parameter adjustment method. Secondly, the sample was divided into four ratings. When setting the upper and lower indices of the trapezoidal membership function, 0.25, 0.5 and 0.75 were taken as the critical values of four equal fractions. For example, there was a fuzzy evaluation of plant height at 0.24 and 0.26, so the fuzzy boundary was set as 0.1, that is, the fuzzy boundary of 0.25 was 0.2-0.3. The trapezoidal membership function of positive indicators of ‘Very tolerant’, ‘Intermediate’, ‘Susceptible’ and ‘Very susceptible’ are shown in formula 5-8 and **Figure 8**. *x*_1_, *x*_2_, *x*_3_, *x*_4_, *x*_5_, *x*_6_ are 0.2, 0.3, 0.45, 0.55, 0.7, and 0.8, respectively. The negative indicators are the opposite.


(5) $$V\left(x\right)=\{\begin{array}{cc} 0, & x<x_{5}\\ \frac{x-x_{5}}{x_{6}-x_{5}}, & x_{5}\leq x\leq x_{6}\\ 1, & x_{6}<x \end{array}$$

(6) $$V\left(x\right)=\{\begin{array}{cc} \frac{x-x_{3}}{x_{4}-x_{3}}, & x_{3}\leq x<x_{4}\\ 1, & x_{4}\leq x<x_{5}\\ \frac{x_{6}-x}{x_{6}-x_{5}}, & x_{5}\leq x<x_{6}\\ 0, & x<x_{3}\;or\;x_{6}<x \end{array}$$

(7) $$V\left(x\right)=\{\begin{array}{cc} \frac{x-x_{1}}{x_{2}-x_{1}}, & x_{1}\leq x<x_{2}\\ 1, & x_{2}\leq x<x_{3}\\ \frac{x_{4}-x}{x_{4}-x_{3}}, & x_{3}\leq x<x_{4}\\ 0, & x<x_{1}\;or\;x_{4}<x \end{array}$$

(8) $$V\left(x\right)=\{\begin{array}{cc} 1, & x<x_{1}\\ \frac{x_{2}-x}{x_{2}-x_{1}}, & x_{1}\leq x\leq x_{2}\\ 0, & x_{2}<x \end{array}$$

5. The fuzzy comprehensive evaluation matrix *r_Pi_* of m indicators of each sample was calculated, and the membership degree $r=\left[\begin{array}{cccc} r_{1} & r_{2} & r_{3} & r_{4} \end{array}\right]$ of each factor to the evaluation grade was calculated according to the membership function constructed by step 4.


(9) $$r_{p_{i}}=\left[\begin{array}{cccc} r_{11} & r_{12} & r_{13} & r_{14}\\ r_{21} & r_{22} & r_{23} & r_{24}\\ \vdots & \vdots & \vdots & \vdots \\ r_{\text{m}1} & r_{\text{m}2} & r_{\text{m}4} & r_{\text{m}4} \end{array}\right]$$

6. For each sample, the fuzzy operator of multiplication and addition was used for fuzzy transformation, and the weight and fuzzy comprehensive evaluation matrix were synthesized into a fuzzy vector *b*. The fuzzy operator of multiplication and addition has a strong degree of synthesis, reflects the weight function obviously, and makes full use of the information of membership degree, which belongs to the weighted average type synthesis operator method.


(10) $$b_{p_{i}}=\sum_{j=1}^{m}\left(w_{j}\cdot r_{j(p_{i})}\right)$$

Where, $w_{j}$ is the weight of the jth evaluation index of *p_i_* sample, $r_{j(p_{i})}$ is the fuzzy comprehensive evaluation matrix of the jth evaluation index of *p_i_* sample, and $b_{p_{i}}$ is the fuzzy vector of *p_i_* sample.

7. The maximum membership principle was used to judge the salt tolerance of the samples.


(11) $$U_{k}\left(p_{i}\right)=\overset{4}{\underset{k=1}{\vee }}b_{k}\left(p_{i}\right)$$

Where, $b_{k}\left(p_{i}\right)$ is the membership degree of $p_{i}$ sample belonging to the kth evaluation level, $U_{k}\left(p_{i}\right)$ is the membership degree of $p_{i}$ sample belonging to the kth evaluation level of the evaluation set, and ∨ is the operation of “taking large” in fuzzy mathematics. In this paper, the scores of 100, 75, 50 and 25 were set to represent four evaluation ratings of ‘very tolerance’, ‘intermediate’, ‘susceptible’ and ‘very susceptible’, respectively.

## 3 Results

### 3.1 Statistical analysis of phenotypic traits

Firstly, the phenotypic traits were statistically analyzed to explore the sensitivity of salt tolerance phenotypic traits to different statistical indicators, so as to determine weighting method of the FCE-E. **Tables 2**, **3** show the coefficient of variation, information entropy and variance contribution rate of TPT and CPT under CK and NaCl conditions, respectively. It could be seen from **Table 2** that the variation coefficient and information entropy of FW, DW and WC were significantly higher than that of chlorophyll and the variation coefficient was above 0.3 after processing of two modes, which showed that different varieties of alfalfa in fresh weight, dry weight and water content of salt tolerance level had great differences and had a greater contribution in salt resistance evaluation. In addition, compared with the CK group, the coefficient of variation of chlorophyll increased, which also showed that chlorophyll had an indicative effect on alfalfa’s salt tolerance, which could be used for salt tolerance identification of alfalfa. It could be seen from **Table 3** that compared with the CK group, the coefficient of variation of the computing phenotypic traits in the NaCl group also changed to different degrees. Due to the influence of salt stress, most indexes increased and they were mainly concentrated in the indicators that represented structural growth, chlorophyll, stress index and biomass, and the information entropy of these indexes accounted for a larger proportion, indicating that these indexes could capture the salt tolerance of alfalfa more sensitively. In addition, the results showed that compared with the TPT, the CPT could find more salt tolerance phenotypes.

**Table 2:** Statistical analysis of the typical phenotypic traits.

Index system	Traits	CK			NaCl		
		CV	H	Var	CV	H	Var
Typical phenotypic traits	FW	0.35	0.34	0.25	0.35	0.29	0.28
	DW	0.33	0.29	0.23	0.40	0.36	0.28
	WC	0.36	0.36	0.25	0.34	0.28	0.28
	CHL	0.05	0.01	0.27	0.17	0.07	0.16

CV is the coefficient of variation, H is the information entropy, and Var is the variance contribution rate.

**Table 3:** Statistical analysis of the computing phenotypic traits.

Index system	Traits	CK			NaCl		
		CV	H	Var	CV	H	Var
Computing phenotypic traits	Height	0.15	0.06	0.06	0.21	0.07	0.06
	Leaf-area	0.15	0.06	0.02	0.25	0.10	0.05
	Volume	0.18	0.08	0.05	0.25	0.10	0.06
	SR680	0.17	0.07	0.06	0.20	0.06	0.07
	PSSRb	0.19	0.10	0.06	0.23	0.08	0.06
	Datt	0.07	0.01	0.06	0.17	0.04	0.07
	mNDVI	0.07	0.01	0.05	0.20	0.06	0.07
	BN	0.09	0.02	0.06	0.16	0.04	0.07
	ARI	0.14	0.05	0.04	0.13	0.03	0.04
	CRI	0.17	0.07	0.05	0.16	0.04	0.06
	Srapa	0.17	0.07	0.06	0.21	0.07	0.07
	TVI	0.10	0.03	0.06	0.08	0.01	0.06
	WI	0.02	0.00	0.05	0.01	0.00	-0.02
	SWSI	0.07	0.01	0.06	0.10	0.01	0.06
	SRVI	0.18	0.09	0.06	0.22	0.07	0.06
	CTR	0.26	0.18	0.05	0.22	0.07	-0.03
	NDVIlut	0.11	0.03	0.06	0.23	0.08	0.07
	NDRI	0.13	0.05	0.06	0.23	0.08	0.06
	NDVI	0.03	0.00	0.06	0.06	0.01	0.06

It was worth noting that in different treatment conditions or different evaluation index systems, the variance contribution rate was relatively average in all phenotypic traits, while the information entropy showed a differential distribution. Information entropy was more able to find the difference of different phenotypic characters of alfalfa under salt stress. Therefore, the FCE-E method in this paper allocated weight proportionally according to the size of information entropy.

### 3.2 Comprehensive evaluation of salt tolerance based on the typical phenotypic traits

**Figure 9:**
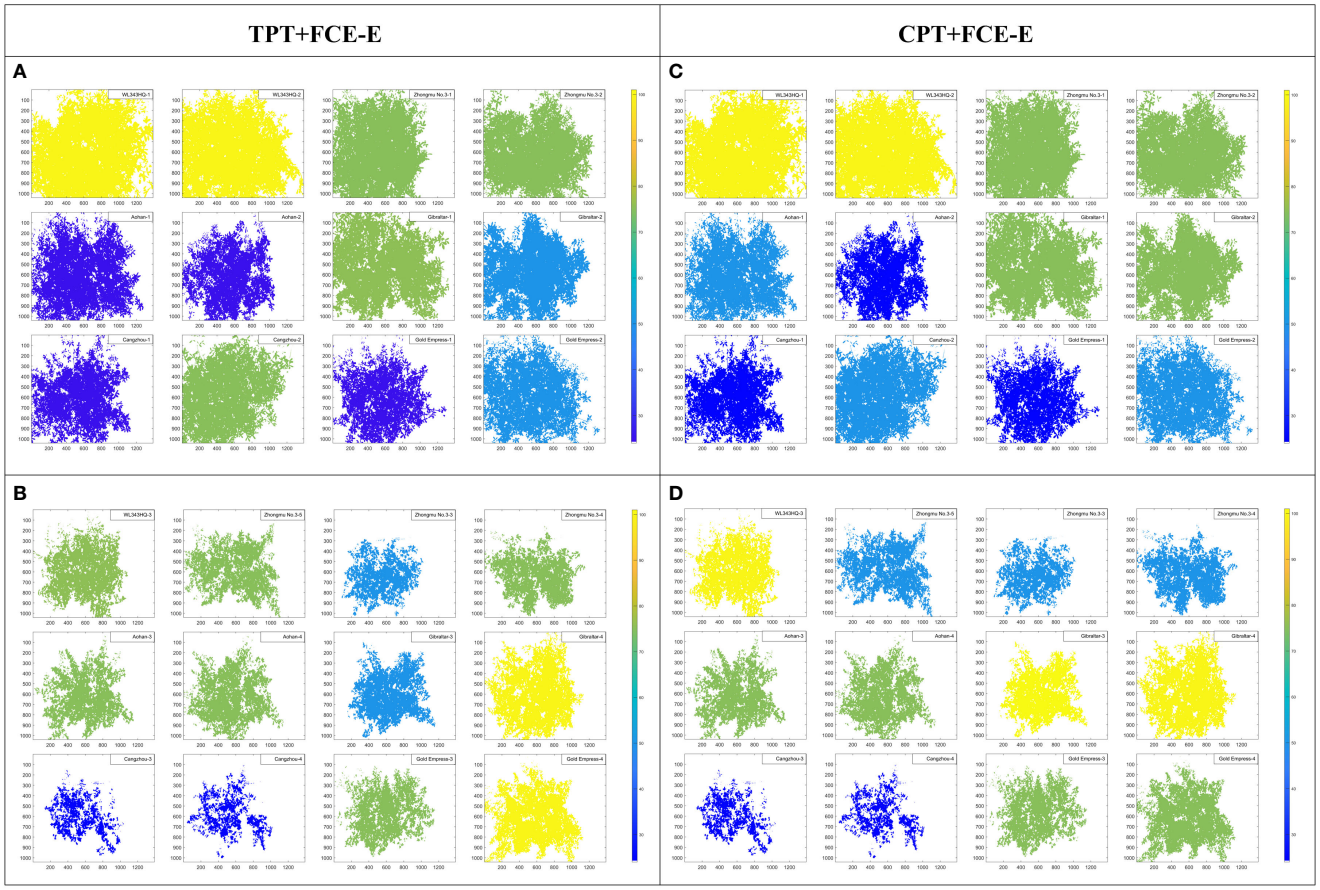
Salt tolerance evaluation results based on FCE-E. **(A, B)** are the results of salt tolerance evaluation were based on typical phenotypic traits. **(A)** for the CK groups, and **(B)** for the NaCl groups. **(C, D)** are the results of salt tolerance evaluation are based on computing phenotypic traits. **(C)** for the CK groups, and **(D)** for the NaCl groups. The scores of 100, 75, 50 and 25 represented the ratings of ‘very tolerance’, ‘intermediate’, ‘susceptible’ and ‘very susceptible’, respectively.

In order to verify the evaluation ability of the multi-index FCE-E model based on the CPT in this paper, the four TPT of FW, DW, WC and CHL were used as the evaluation index system, and the salt tolerance evaluation of alfalfa was carried out by the FCE-E method. The alfalfa was divided into four grades, and the results are shown in **Figures 9A, B**. Under NaCl condition, the growth of other alfalfa varieties was affected, except that the salt tolerance rating of Aohan, Gibraltar and Gold Empress were improved compared with the CK condition. Aohan had a poor rating under CK conditions, on the contrary, it was rated as intermediate salt tolerance under NaCl, indicating that the variety was not suitable for cultivation in undamaged land and was more suitable for cultivation and improvement promotion in saline-alkali land. Cangzhou had a poor rating under salt stress conditions, indicating that the variety of alfalfa was not suitable for cultivation on saline-alkali land.

### 3.3 Comprehensive evaluation of salt tolerance based on the computing phenotypic traits

Using the CPT as the multi-index evaluation system of salt tolerance, the FCE-E method was used to construct a non-destructive evaluation model of salt tolerance of alfalfa. Alfalfa was divided into four grades (**Figures 9C, D**) and compared with the evaluation result of salt tolerance based on TPT (in section 3.2). Under the condition of salt stress (**Figure 9D**), WL343HQ and Gibraltar were rated as salt-tolerant alfalfa varieties, Aohan and Gold Empress were rated as intermediate salt-tolerant alfalfa varieties, all Zhongmu No. 3 were rated as susceptible salt-sensitive alfalfa varieties, and Cangzhou were all highly salt susceptible varieties. Therefore, 3 highly tolerant, 4 intermediate, 3 susceptible and 2 highly susceptible materials were screened in this paper.

Compared to section 3.2, a total of 66.67% had consistent results. Among them, in the CK group, there were 75% consistent results; there were 58.33% consistent results in the NaCl group. Affected by salt stress, the physical and chemical characteristics of alfalfa would change in varying degrees. Compared with TPT, the CPT could find more salt-sensitive information. Therefore, the consistency rate of the two results in the NaCl group is reduced.

### 3.4 Comprehensive evaluation of salt tolerance by PCA method

**Figure 10:**
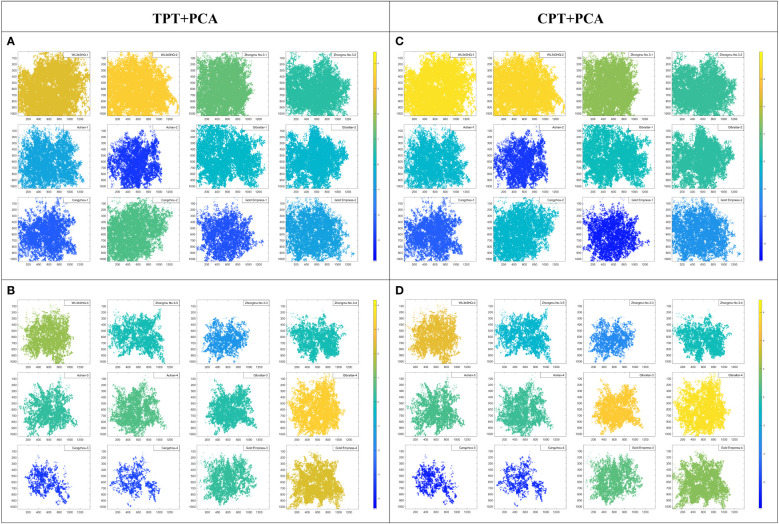
Salt tolerance evaluation results based on PCA. **(A, B)** are the results of salt tolerance evaluation were based on typical phenotypic traits. **(A)** for the CK groups, and **(B)** for the NaCl groups. **(C, D)** are the results of salt tolerance evaluation are based on computing phenotypic traits. **(C)** for the CK groups, and **(D)** for the NaCl groups.

To further explore the reliability of the multi-index FCE-E model, the PCA method was compared with the FCE-E method. The results are shown in **Table 4**. In the CK group, the first principal component accounted for 87.03% based on the PCA results of TPT, and 85.43% based on the CPT. In the NaCl group, the first principal component based on TPT accounted for 88.19%, and the first principal component based on the CPT accounted for 79.43%. The first principal component had good explanatory power, so only the first principal component was extracted as the composite score. The results of **Figures 9**, **10** showed that the results of PCA were basically consistent with the results of the multi-index FCE-E, but the results of PCA could only rank the salt tolerance of the samples, and could not determine the salt tolerance level. Therefore, the results of the multi-index FCE-E model are more reliable and intuitive.

**Table 4:** PCA salt tolerance rating results of CK and NaCl groups.

Treatment	Sample	TPT	Rank	CPT	Rank	Treatment	Sample	TPT	Rank	CPT	Rank
CK	WL343HQ-1	2.66	2	6.98	1	NaCl	WL343HQ-3	-1.45	3	-3.83	3
	WL343HQ-2	3.45	1	6.56	2		Zhongmu No.3-3	1.49	10	3.35	10
	Zhongmu No.3-1	1.26	3	2.95	3		Zhongmu No.3-4	0.06	8	0.53	8
	Zhongmu No.3-2	0.57	5	1.16	4		Zhongmu No.3-5	0.07	9	1.24	9
	Aohan-1	-0.64	8	-0.33	8		Aohan-3	-0.26	6	-0.61	6
	Aohan-2	-2.85	12	-5.40	11		Aohan-4	-0.66	4	-0.35	7
	Gibraltar-1	-0.01	6	0.25	6		Gibraltar-3	-0.10	7	-4.98	2
	Gibraltar-2	-0.09	7	0.88	5		Gibraltar-4	-3.21	1	-5.87	1
	Cangzhou-1	-2.23	10	-4.26	10		Cangzhou-3	3.44	12	6.91	12
	Cangzhou-2	1.01	4	-0.32	7		Cangzhou-4	3.18	11	6.44	11
	Gold Empress-1	-2.40	11	-6.30	12		Gold Empress-3	-0.30	5	-0.96	5
	Gold Empress-2	-0.73	9	-2.19	9		Gold Empress-4	-2.26	2	-1.87	4

## 4 Discussion

The problem of soil salinization is becoming more and more serious worldwide, and screening and promoting salt-tolerant forage is the main way to improve and rationally use salinized soils (Singh et al., 2018). At present, the salt tolerance of maize (Fortmeier and Schubert, 1995), rice (Kumar et al., 2012) and other major crops has been deeply studied. Alfalfa, as the most widely planted and salt-tolerant forage, has high research value, so it has been studied in term of growth monitoring, salt tolerance mechanism and salt tolerance screening of alfalfa. However, for the non-destructive screening of alfalfa salt tolerance, there is still a lack of an accurate and systematic salt tolerance evaluation method. Miao et al. comprehensively evaluated the survival rate, plant height, number of green leaves, number of wilted leaves, number of branches and aboveground biomass of alfalfa seedlings by the membership function method (Lihong et al., 2016). Wu et al. explored the genetic diversity of alfalfa germplasm resources by using morphological indexes, agronomic traits and quality traits. The results showed that the agronomic traits of different alfalfa germplasm had the largest variation, followed by morphological traits and quality traits (Xinming et al., 2018). In this paper, based on previous studies, the CPT of alfalfa was constructed using HSI data and LiDAR data. The multi-index FCE-E model was used to evaluate the salt tolerance of 24 alfalfa materials, so as to obtain highly salt-tolerant and highly salt-susceptible varieties.

### 4.1 Advantages of computing phenotypic traits in comprehensive evaluation of salt tolerance

Combining multiple indicators to screen for salt-tolerant materials is the most reliable research method today (Hu et al., 2018). However, the traditional method of obtaining phenotypic traits has the disadvantages of low measurement flux, time-consuming and labor-consuming, and data acquisition is difficult, especially for large-scale measurements. At present, the traditional phenotypic traits can no longer meet the needs of the rapidly developing plant stress resistance research, which seriously hinders the excavation of alfalfa salt-tolerant germplasm, so the high-throughput CPT have emerged (Wu et al., 2021). A series of changes occurred in its internal physiological components and external morphological structure after alfalfa was treated with salt stress.

**Figure 11:**
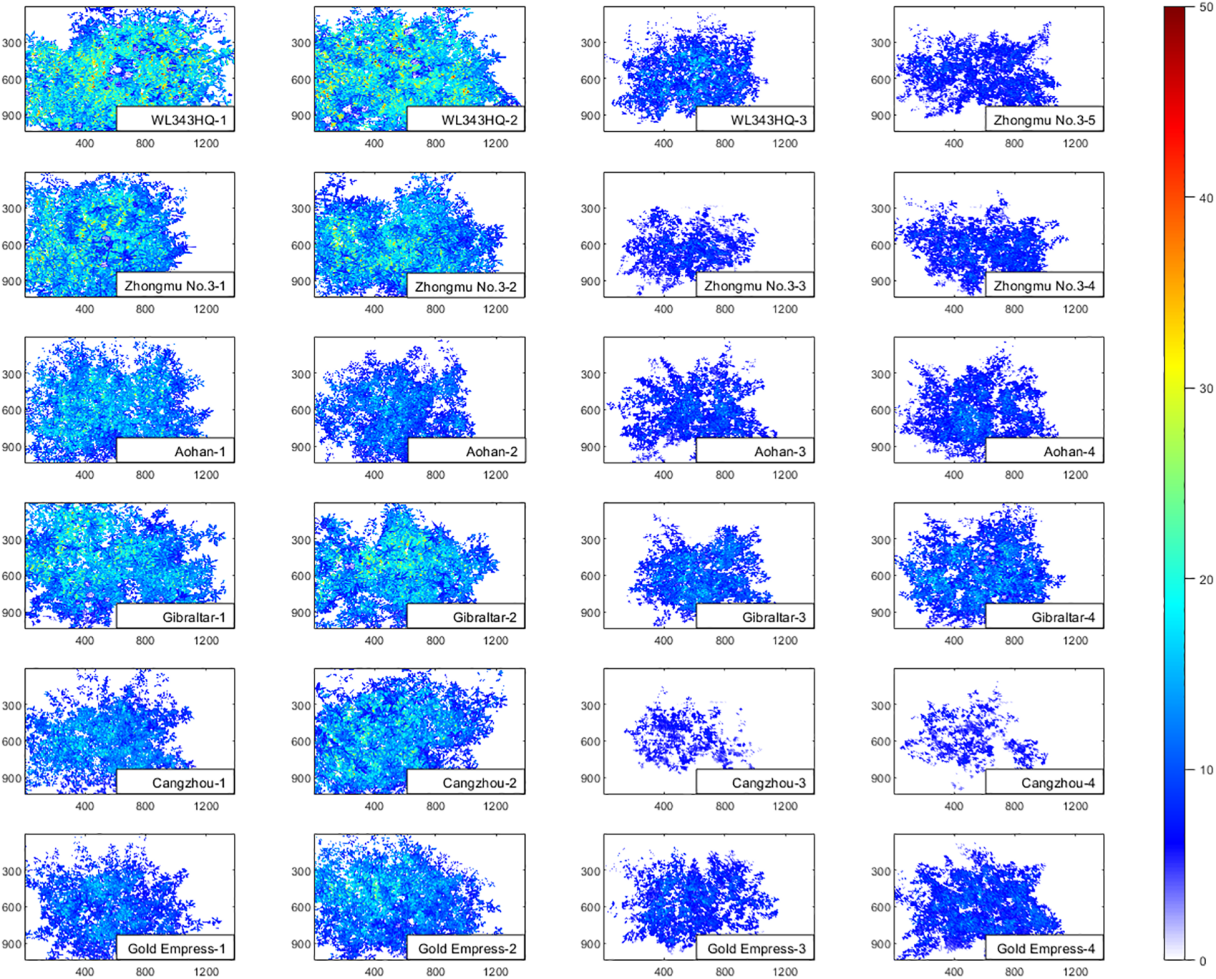
The schematic diagram of SR680 vegetation index of alfalfa in CK groups and NaCl groups. The left two columns are CK groups, and the right two columns are NaCl groups.

Firstly, different spectral bands of HSI can capture the differences in various pigments, moisture content and biomass in alfalfa. Hyperspectral imaging technology was used to supplement and expand the TPT. For example, the contents of pigments such as anthocyanin and salt stress index of alfalfa were non-destructively extracted by using spectral phenotypic traits compared with the measured TPT. Taking the chlorophyll phenotypic trait SR680 vegetation index of alfalfa as an example, **Figure 11** visually shows the difference between alfalfa in CK groups and NaCl groups. It could be seen from the figure that the chlorophyll content of alfalfa in the CK groups were significantly higher than that in the NaCl groups. In the single-basin canopy scale, the chlorophyll content in the leaf center was higher than that in the leaf edge.

Secondly, LiDAR technology can accurately obtain its 3D structure information. In order to evaluate the accuracy of the structural phenotypic traits extracted from the LiDAR data, we conducted a correlation analysis between the number of pixels representing the alfalfa area extracted from the HSI data and the canopy leaf area extracted from the LiDAR data. The correlation coefficient (R2) was found to be 0.7434, indicating a good quality of the canopy leaf area extracted from the LiDAR data.

Finally, it could be found from **Figure 11** that the CPT combine spectral and spatial dimension information, which can sensitively capture the differences of alfalfa under salt stress, and contribute to more comprehensive and lower cost evaluation of salt tolerance and germplasm screening of alfalfa. The results of salt-tolerant germplasm screening of alfalfa based on the CPT were similar to those of the TPT, and the results of the former were more consistent with those of manual screening, which provided a basis for automatic, low-difficulty and high-time-based salt-tolerance evaluation and breeding (**Figure 9**). Since more components were not detected in this material, they were not reflected in the TPT. More typical phenotypic traits should be added in future comprehensive rating experiments as an evaluation reference.

### 4.2 Portability and sensitivity of the multi-index FCE-E model

In the CK and NaCl groups, different phenotypic traits were affected by the growth environment to different degrees. Salt stress can hinder the growth of alfalfa, reduce the rate of leaf differentiation, slow down photosynthesis, and lead to different degrees of decrease or increase in phenotypic traits. The multi-index FCE-E model in this paper determined the weight of each trait in the evaluation of salt tolerance according to the information entropy of each factor. The smaller the information entropy of each trait was, the smaller the weight and the contribution to the salt tolerance rating would be. The PCA method determined the weight according to the variance contribution rate of each trait. The smaller the variance contribution rate was, the smaller the assigned weight and the smaller the contribution to salt tolerance rating would be. According to **Tables 2**, **3**, the variance contribution rate of each phenotypic trait in the CK and NaCl groups were relatively balanced, and the information entropy presented a differential distribution. Therefore, compared with the variance contribution rate, the information entropy could better capture the response difference of phenotypic traits under salt stress. On the one hand, the entropy weight method could objectively assign weights to different phenotypic traits. On the other hand, it could adapt to the influence of different phenotypic traits on the salt tolerance evaluation model in different treatment conditions and increase the portability of the model. In addition, the FCE-E method in this paper adopted the method of adaptive adjustment when setting the membership function parameters, which also increases the portability of the model.

**Figure 12:**
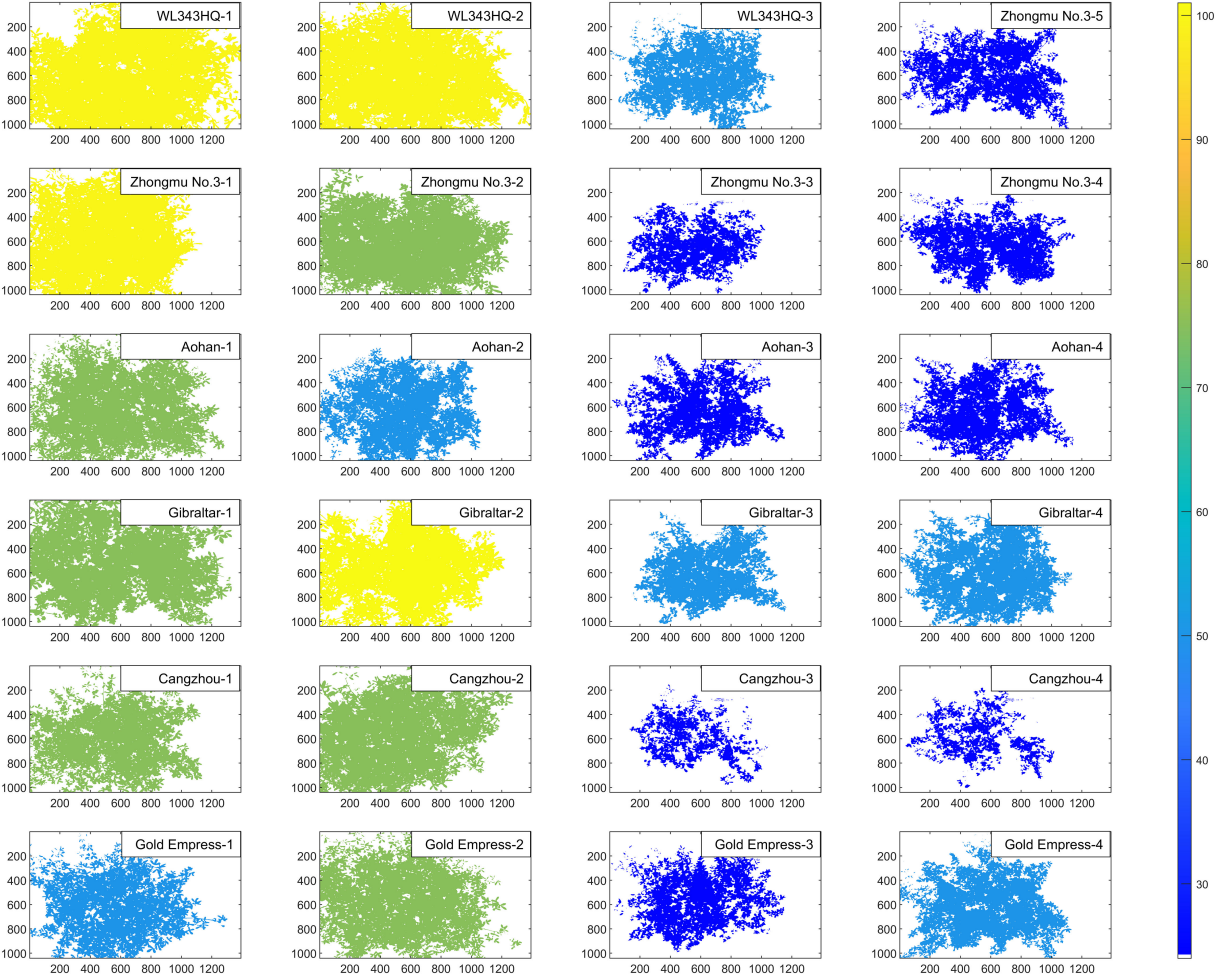
Results of salt tolerance evaluation of 24 samples using the FCE-E method. The left two columns are CK groups, and the right two columns are NaCl groups.

To test the sensitivity of the multi-index FCE-E model in evaluating the salt tolerance of alfalfa, the CK and NaCl groups were combined, and the multi-index FCE-E comprehensive ratings were performed on the same sample set. The results of salt tolerance evaluation of 24 samples (**Figure 12**) showed that the growth status of all varieties of the NaCl group was worse than that of the CK group, indicating that the multi-index FCE-E model based on the CPT could effectively characterize the salt tolerance of alfalfa. Therefore, according to section 3.3 (**Figures 9C, D**), the salt tolerance of 12 sample materials in the NaCl group was determined, and 3 highly salt-tolerant, 4 intermediate, 3 susceptible and 2 highly susceptible materials were obtained. In addition, as shown in **Figure 9**, WL343HQ had better growth performance in both the CK and NaCl groups, indicating that WL343HQ had more stable salt tolerance and could be cultivated and promoted in more regions.

### 4.3 Comparison of the multi-index FCE-E and the PCA

The results of the comprehensive evaluation of salt tolerance of alfalfa by the multi-index FCE-E method and the PCA method proposed in this paper (**Figures 9**, **10**) showed that the two methods got relatively consistent results, and the multi-index FCE-E method could select salt-tolerant and highly salt-susceptible materials more intuitively, while the PCA method could only get the ranking of salt tolerance degree of alfalfa, and the selection of salt-tolerance materials could not be directly derived from the results. In addition, subjective factors are involved in the determination of the contribution rate of principal components in the comprehensive evaluation of salt tolerance using the PCA method.

The multi-index FCE-E method proposed in this paper was based on the probability and statistical theory of fuzzy mathematics, and the method could objectively evaluate the salt tolerance of alfalfa by using adaptive parameter settings. The results of multi-index FCE-E were the rating of salinity tolerance of alfalfa, the results of which intuitively gave the corresponding level in all materials. The better the result was, the better its growth status and tolerance to salt stress would be. Moreover, the results of salt tolerance evaluation based on the two evaluation index systems were similar, so the growth status of alfalfa could be evaluated and its salt tolerance could be judged by nondestructive CPT.

### 4.4 Application of the FCE-E method in large-scale breeding programs

For plant breeders, the FCE-E method can help them make many improvements in large-scale breeding programs. First, plant breeders can evaluate the salt tolerance of different alfalfa varieties based on this method. According to this method, the high-quality varieties with the highest salt tolerance score of alfalfa were determined, and the varieties for in-depth breeding or commercial promotion were preliminarily screened. Secondly, this method can help plant breeders determine more important phenotypic traits in different batches of alfalfa. By analyzing the weights assigned to each trait, breeders can explore potential breeding advantages and defects, and focus on the most critical traits, which can improve the accuracy and efficiency of breeding. Finally, the use of LiDAR and HSI data can shorten the breeding time and reduce the breeding cost. Through non-destructive measurement, breeders can simultaneously evaluate large areas of alfalfa, thereby quickly optimizing breeding strategies. In general, this method can help breeders select the best varieties and breeding strategies through systematic evaluation of physiology, biochemistry and structure, and improve the efficiency and effectiveness of large-scale breeding programs.

## 5 Conclusions

Through the improved fuzzy comprehensive evaluation method, the salt tolerance of 6 alfalfa varieties was comprehensively evaluated in this study, and the following main conclusions were obtained: Firstly, the multi-index FCE-E method proposed in this paper can accurately evaluate the response ability of alfalfa to salt stress. Secondly, this method combines entropy method and fuzzy comprehensive evaluation method to capture the differential performance of alfalfa phenotypic traits in response to salt stress in a more objective and sensitive way. In addition, the method uses the calculated phenotypic traits as the data source, which can more quickly and comprehensively find the differences and changes of alfalfa physical and chemical parameters and structural parameters.

## Data availability statement

The original contributions presented in the study are included in the article/supplementary materials. Further inquiries can be directed to the corresponding author.

## Author contributions

AZ and JZ designed the research. All authors performed the experiments and analyzed the data. AZ and JZ initially drafted the manuscript with inputs from other authors. All the authors read and approved the final manuscript. All authors contributed to the article and approved the submitted version.

## References

[B1] Al-Tamimi N. Langan P. Bernad V. Walsh J. Mangina E. Negrao S. (2022). Capturing crop adaptation to abiotic stress using image-based technologies. Open Biol. 12 (6), 210353. doi: 10.1098/rsob.210353 35728624 PMC9213114

[B2] Aparicio N. Villegas D. Araus J. L. Casadesus J. Royo C. (2002). Relationship between growth traits and spectral vegetation indices in durum wheat. Crop Sci. 42, 1547–1555. doi: 10.2135/cropsci2002.1547

[B3] Barnes E. Clarke T. R. Richards S. E. Colaizzi P. Haberland J. Kostrzewski M.. (2000). Coincident detection of crop water stress, nitrogen status, and canopy density using ground based multispectral data. Environ. Sci. 1619, 1–15.

[B4] Blackburn G. A. (2010). Spectral indices for estimating photosynthetic pigment concentrations: A test using senescent tree leaves. Int. J. Remote Sens. 19 (4), 657–675. doi: 10.1080/014311698215919

[B5] Broge N. H. Leblanc E. (2001). Comparing prediction power and stability of broadband and hyperspectral vegetation indices for estimation of green leaf area index and canopy chlorophyll density. Remote Sens. Environ. 76 (2), 156–172. doi: 10.1016/S0034-4257(00)00197-8

[B6] Brook A. Tal Y. Markovich O. Rybnikova N. (2021). Canopy Volume as a Tool for Early Detection of Plant Drought and Fertilization Stress: Banana plant fine-phenotype. bioRxiv doi: 10.1101/2021.03.04.433868

[B7] Buschmann C. Nagel E. (2007). *In vivo* spectroscopy and internal optics of leaves as basis for remote sensing of vegetation. Int. J. Remote Sens. 14 (4), 711–722. doi: 10.1080/01431169308904370

[B8] Carter G. A. (2007). Ratios of leaf reflectances in narrow wavebands as indicators of plant stress. Int. J. Remote Sens. 15 (3), 697–703. doi: 10.1080/01431169408954109

[B9] Chunjiang Z. (2019). Big data of plant phenomics and its research progress. J. Agric. Big Data 1 (02), 5–18. doi: 10.19788/j.issn.2096-6369.190201

[B10] Datt B. (2010). Visible/near infrared reflectance and chlorophyll content in Eucalyptus leaves. Int. J. Remote Sens. 20 (14), 2741–2759. doi: 10.1080/014311699211778

[B11] Du Y.-W. Wang S.-S. Wang Y.-M. (2019). Group fuzzy comprehensive evaluation method under ignorance. Expert Syst. Appl. 126, 92–111. doi: 10.1016/j.eswa.2019.02.006

[B12] Duan J. Li J. Guo S. Kang Y. (2008). Exogenous spermidine affects polyamine metabolism in salinity-stressed Cucumis sativus roots and enhances short-term salinity tolerance. J. Plant Physiol. 165 (15), 1620–1635. doi: 10.1016/j.jplph.2007.11.006 18242770

[B13] Duo L. Shuang B. Zhijie L. Qingshan Y. Xuebin Q. Wei G.. (2021). A review on the saline-alkaline tolerance of alfalfa (Medicago sativa L.). J. OF Biol. 38 (01), 98–101+105. doi: 10.3969/j.issn.2095-1736.2021.01.098

[B14] El-Hendawy S. Al-Suhaibani N. Elsayed S. Alotaibi M. Hassan W. Schmidhalter U. (2019a). Performance of optimized hyperspectral reflectance indices and partial least squares regression for estimating the chlorophyll fluorescence and grain yield of wheat grown in simulated saline field conditions. Plant Physiol. Biochem. 144, 300–311. doi: 10.1016/j.plaphy.2019.10.006 31605962

[B15] El-Hendawy S. E. Al-Suhaibani N. A. Hassan W. M. Dewir Y. H. Elsayed S. Al-Ashkar I.. (2019b). Evaluation of wavelengths and spectral reflectance indices for high-throughput assessment of growth, water relations and ion contents of wheat irrigated with saline water. Agric. Water Manage. 212, 358–377. doi: 10.1016/j.agwat.2018.09.009

[B16] El-Hendawy S. Dewir Y. H. Elsayed S. Schmidhalter U. Al-Gaadi K. Tola E.. (2022). Combining hyperspectral reflectance indices and multivariate analysis to estimate different units of chlorophyll content of spring wheat under salinity conditions. Plants 11 (3), 456. doi: 10.3390/plants11030456 35161437 PMC8839343

[B17] Fortmeier R. Schubert S. (1995). Salt tolerance of maize ( Zea mays L.) : The role of sodium exclusion. Plant Cell Environ. 18 (9), 1041–1047. doi: 10.1111/j.1365-3040.1995.tb00615.x

[B18] Gitelson A. A. Merzlyak M. N. Chivkunova O. B. (2001). Optical properties and nondestructive estimation of anthocyanin content in plant leaves. Photochem. Photobiol. 74 (1), 38–45. doi: 10.1562/0031-8655(2001)0740038OPANEO2.0.CO2 11460535

[B19] Gitelson A. A. Zur Y. Chivkunova O. B. Merzlyak M. N. (2002). Assessing carotenoid content in plant leaves with reflectance spectroscopy. Photochem. Photobiol. 75 (3), 272–281. doi: 10.1562/0031-8655(2002)0750272ACCIPL2.0.CO2 11950093

[B20] Guiza M. Benabdelrahim M. A. Brini F. Haddad M. Saibi W. (2022). Assessment of alfalfa (Medicago sativa L.) cultivars for salt tolerance based on yield, growth, physiological, and biochemical traits. J. Plant Growth Regul. 41 (8), 3117–3126. doi: 10.1007/s00344-021-10499-9

[B21] Hamzeh S. Naseri A. A. AlaviPanah S. K. Mojaradi B. Bartholomeus H. M. Clevers J. G. P. W.. (2013). Estimating salinity stress in sugarcane fields with spaceborne hyperspectral vegetation indices. Int. J. Appl. Earth Observation Geoinformation 21, 282–290. doi: 10.1016/j.jag.2012.07.002

[B22] Hanin M. Ebel C. Ngom M. Laplaze L. Masmoudi K. (2016). New insights on plant salt tolerance mechanisms and their potential use for breeding. Front. Plant Sci. 7. doi: 10.3389/fpls.2016.01787 PMC5126725 27965692

[B23] Hongyan L. Zongli W. (2007). Descriptors and Data Standard for Medic (Medicago Linn.) (China Agriculture Press).

[B24] Houle D. Govindaraju D. Omholt S. (2010). Phenomics: the next challenge. Nat. Rev. Genet. 11 (12), 855–866. doi: 10.1038/nrg2897 21085204

[B25] Hu Y. Schmidhalter U. (2023). Opportunity and challenges of phenotyping plant salt tolerance. Trends Plant Sci. 28 (5), 552–566. doi: 10.1016/j.tplants.2022.12.010 36628656

[B26] Hu D. Wu D. You J. He Y. Qian W. (2018). Principal component analysis and comprehensive evaluation on salt tolerance related traits in Brassica napus L. Botanical Res. 07 (02), 101–112. doi: 10.12677/br.2018.72014

[B27] Hunt E. R. Doraiswamy P. C. McMurtrey J. E. Daughtry C. S. T. Perry E. M. Akhmedov B. (2013). A visible band index for remote sensing leaf chlorophyll content at the canopy scale. Int. J. Appl. Earth Observation Geoinformation 21, 103–112. doi: 10.1016/j.jag.2012.07.020

[B28] Ismail A. M. Horie T. (2017). Genomics, physiology, and molecular breeding approaches for improving salt tolerance. Annu. Rev. Plant Biol. 68 (1), 405–434. doi: 10.1146/annurev-arplant-042916-040936 28226230

[B29] Jimenez-Berni J. A. Deery D. M. Rozas-Larraondo P. Condon A. G. Rebetzke G. J. James R. A.. (2018). High throughput determination of plant height, ground cover, and above-ground biomass in wheat with LiDAR. Front. Plant Sci. 9. doi: 10.3389/fpls.2018.00237 PMC5835033 29535749

[B30] Jin S. Sun X. Wu F. Su Y. Li Y. Song S.. (2021a). Lidar sheds new light on plant phenomics for plant breeding and management: Recent advances and future prospects. ISPRS J. Photogrammetry Remote Sens. 171, 202–223. doi: 10.1016/j.isprsjprs.2020.11.006

[B31] Jin X. Zarco-Tejada P. J. Schmidhalter U. Reynolds M. P. Hawkesford M. J. Varshney R. K.. (2021b). High-throughput estimation of crop traits: A review of ground and aerial phenotyping platforms. IEEE Geosci. Remote Sens. Magazine 9 (1), 200–231. doi: 10.1109/mgrs.2020.2998816

[B32] Kasim N. Qingdong S. Jingzhe W. Sawut R. Nurmemet I. Isak G. (2017). Estimation of spring wheat chlorophyll content based on hyperspectral features and PLSR model. Trans. Chin. Soc. Agric. Eng. (Transactions CSAE) 33 (22), 208–216. doi: 10.11975/j.issn.1002-6819.2017.22.027

[B33] Kumar R. Mustafiz A. Sahoo K. K. Sharma V. Samanta S. Sopory S. K.. (2012). Functional screening of cDNA library from a salt tolerant rice genotype Pokkali identifies mannose-1-phosphate guanyl transferase gene (OsMPG1) as a key member of salinity stress response. Plant Mol. Biol. 79 (6), 555–568. doi: 10.1007/s11103-012-9928-8 22644442

[B34] Li J. Schachtman D. P. Creech C. F. Wang L. Ge Y. Shi Y. (2022). Evaluation of UAV-derived multimodal remote sensing data for biomass prediction and drought tolerance assessment in bioenergy sorghum. Crop J. 10 (5), 1363–1375. doi: 10.1016/j.cj.2022.04.005

[B35] Lihong M. Qinghua Y. Yu W. (2016). Evaluation on salt tolerance of 41 alfalfa varieties at seedling stage. Seed 35 (04), 81–84. doi: 10.16590/j.cnki.1001-4705.2016.04.081

[B36] Luther J. E. Carroll A. L. (1999). Development of an index of balsam fir vigor by foliar spectral reflectance. Remote Sens. Environ. 69 (3), 241–252. doi: 10.1016/S0034-4257(99)00016-4

[B37] M L.Y. H G.Q. B W. N Q.H. Z W. D. X X.K.. (2021). Current status and prospect of threedimensional dynamic monitoring of natural resources based on LiDAR. Natl. Remote Sens. Bull. 25 (1), 381–402. doi: 10.11834/jrs.20210351

[B38] Munns R. Tester M. (2008). Mechanisms of salinity tolerance. Annu. Rev. Plant Biol. 59 (1), 651–681. doi: 10.1146/annurev.arplant.59.032607.092911 18444910

[B39] Penglei L. Xiao Z. Wenhui W. Hengbiao Z. Xia Y. Yan Z.. (2021). Assessment of terretrial laser scanning and hypersprctral remote sensing for the estimation of rice grain yield. Scientia Agricultura Sin. 54 (14), 2965–2976. doi: 10.3864/j.issn.0578-1752.2021.14.004

[B40] Penuelas J. Pinol J. Ogaya R. Filella I. (2010). Estimation of plant water concentration by the reflectance Water Index WI (R900/R970). Int. J. Remote Sens. 18 (13), 2869–2875. doi: 10.1080/014311697217396

[B41] Post C. J. DeGloria S. D. Cherney J. H. Mikhailova E. A. (2007). Spectral measurements of alfalfa/grass fields related to forage properties and species composition. J. Plant Nutr. 30 (11), 1779–1789. doi: 10.1080/01904160701626951

[B42] Reddy P. Guthridge K. M. Panozzo J. Ludlow E. J. Spangenberg G. C. Rochfort S. J. (2022). Near-infrared hyperspectral imaging pipelines for pasture seed quality evaluation: an overview. Sensors (Basel) 22 (5), 1981. doi: 10.3390/s22051981 35271127 PMC8914962

[B43] Robati M. Rezaei F. (2021). Evaluation and ranking of urban sustainability based on sustainability assessment by fuzzy evaluation model. Int. J. Environ. Sci. Technol. 19 (1), 625–650. doi: 10.1007/s13762-021-03128-1

[B44] Roy S. J. Negrao S. Tester M. (2014). Salt resistant crop plants. Curr. Opin. Biotechnol. 26, 115–124. doi: 10.1016/j.copbio.2013.12.004 24679267

[B45] Saric R. Nguyen V. D. Burge T. Berkowitz O. Trtilek M. Whelan J.. (2022). Applications of hyperspectral imaging in plant phenotyping. Trends Plant Sci. 27 (3), 301–315. doi: 10.1016/j.tplants.2021.12.003 34998690

[B46] Shangpeng S. Changying L. H. P. A. Yu J. Rui X. S. R. J.. (2018). In-field high throughput phenotyping and cotton plant growth analysis using LiDAR. Front. Plant Sci. 9. doi: 10.3389/fpls.2018.00016 PMC5786533 29403522

[B47] Shaochen L. Aiwu Z. Xizhen Z. Zhiqiang Y. Mengnan L. (2023). Phenotype leaf angles of corn seedlings using computational methods. Laser Optoelectronics Prog. 60 (02), 71–79.

[B48] Shaohua Z. Jianzhao D. Li H. Yuhang J. Schulthess U. Lashkari A.. (2022). Wheat yield estimation from UAV platform based on multi-modal remote sensing data fusion. Acta Agronomica Sin. 48 (07), 1746–1760.

[B49] Sims D. A. Gamon J. A. (2002). Relationships between leaf pigment content and spectral reflectance across a wide range of species, leaf structures and developmental stages. Remote Sens. Environ. 81, 337–354. doi: 10.1016/S0034-4257(02)00010-X

[B50] Singh A. P. Bhutia P. (2022). High throughput phenotyping in crop improvement programmes. Readers Shelf 18 (09), 29–32.

[B51] Singh A. Jones S. Ganapathysubramanian B. Sarkar S. Mueller D. Sandhu K.. (2021). Challenges and opportunities in machine-augmented plant stress phenotyping. Trends Plant Sci. 26 (1), 53–69. doi: 10.1016/j.tplants.2020.07.010 32830044

[B52] Singh V. Singh A. P. Bhadoria J. Giri J. Singh J. T V. V.. (2018). Differential expression of salt-responsive genes to salinity stress in salt-tolerant and salt-sensitive rice (Oryza sativa L.) at seedling stage. Protoplasma 255 (6), 1667–1681. doi: 10.1007/s00709-018-1257-6 29740721

[B53] Song P. Wang J. Guo X. Yang W. Zhao C. (2021). High-throughput phenotyping: Breaking through the bottleneck in future crop breeding. Crop J. 9 (3), 633–645. doi: 10.1016/j.cj.2021.03.015

[B54] Sun Z. Li Q. Jin S. Song Y. Xu S. Wang X.. (2022). Simultaneous prediction of wheat yield and grain protein content using multitask deep learning from time-series proximal sensing. Plant Phenomics 2022, 13. doi: 10.34133/2022/9757948 PMC8988204 35441150

[B55] Thenkabail P. S. Mariotto I. Gumma M. K. Middleton E. M. Landis D. R. Huemmrich K. F. (2013). Selection of hyperspectral narrowbands (HNBs) and composition of hyperspectral twoband vegetation indices (HVIs) for biophysical characterization and discrimination of crop types using field reflectance and Hyperion/EO-1 data. IEEE J. Selected Topics Appl. Earth Observations Remote Sens. 6 (2), 427–439. doi: 10.1109/jstars.2013.2252601

[B56] Tilly N. Aasen H. Bareth G. (2015). Fusion of plant height and vegetation indices for the estimation of barley biomass. Remote Sens. 7 (9), 11449–11480. doi: 10.3390/rs70911449

[B57] Tilly N. Hoffmeister D. Cao Q. Lenz-Wiedemann V. Miao Y. Bareth G. (2013). Precise plant height monitoring and biomass estimation with Terrestrial Laser Scanning in paddy rice. ISPRS Ann. Photogrammetry Remote Sens. Spatial Inf. Sci. II-5/W2, 295–300. doi: 10.5194/isprsannals-II-5-W2-295-2013

[B58] Tmušić G. Manfreda S. Aasen H. James M. R. Gonçalves G. Ben-Dor E.. (2020). Current practices in UAS-based environmental monitoring. Remote Sens. 12 (6), 1001. doi: 10.3390/rs12061001

[B59] Tucker C. J. (1977). Spectral estimation of grass canopy variables. Remote Sens. Environ. 6 (1), 11–26. doi: 10.1016/0034-4257(77)90016-5

[B60] Tucker C. J. (1979). Red and photographic infrared l,lnear combinations for monitoring vegetation. Remote Sens. Environ. 8 (2), 127–150. doi: 10.1016/0034-4257(79)90013-0

[B61] Ullah S. Schlerf M. Skidmore A. K. Hecker C. (2012). Identifying plant species using mid-wave infrared (2.5–6μm) and thermal infrared (8–14μm) emissivity spectra. Remote Sens. Environ. 118, 95–102. doi: 10.1016/j.rse.2011.11.008

[B62] Wang D. Wilson C. Shannon M. C. (2010). Interpretation of salinity and irrigation effects on soybean canopy reflectance in visible and near-infrared spectrum domain. Int. J. Remote Sens. 23 (5), 811–824. doi: 10.1080/01431160110070717

[B63] Wu X. Feng H. Wu D. Yan S. Zhang P. Wang W.. (2021). Using high-throughput multiple optical phenotyping to decipher the genetic architecture of maize drought tolerance. Genome Biol. 22 (1), 185. doi: 10.1186/s13059-021-02377-0 34162419 PMC8223302

[B64] Wu C. Niu Z. Tang Q. Huang W. (2008). Estimating chlorophyll content from hyperspectral vegetation indices: Modeling and validation. Agric. For. Meteorology 148 (8-9), 1230–1241. doi: 10.1016/j.agrformet.2008.03.005

[B65] Xiangfeng Z. Xiaorong Y. Ziwei J. (2018). Research progress of salt tolerance evaluation in plants and tolerance evaluation strategy. J. Biol. 35 (06), 91–94. doi: 10.3969/j.issn.2095-1736.2018.06.091

[B66] Xiaofeng C. Keqiang Y. Yanru Z. Haihui Z. (2020). Current status of high-throughput plant phenotyping for abiotic stress by imaging spectroscopy: a review. Spectrosc. Spectral Anal. 40 (11), 3365–3372. doi: 10.3964/j.issn.1000-0593(2020)11-3365-08

[B67] Xinming W. Pu G. Huiwu C. Zhihong F. Yonghong S. Yunqi W.. (2018). Diversity Analysis of Phenotypic Traits and Quality Characteristics of Alfalfa( Medicago sativa) introducted from abroad Germplasm Resources. J. Plant Genet. Resour. 19 (01), 103–111. doi: 10.13430/j.cnki.jpgr.2018.01.012

[B68] Xu X. Nie C. Jin X. Li Z. Zhu H. Xu H.. (2021). A comprehensive yield evaluation indicator based on an improved fuzzy comprehensive evaluation method and hyperspectral data. Field Crops Res. 270. doi: 10.1016/j.fcr.2021.108204

[B69] Zadeh. L. A. (1965). Fuzzy sets. Inf. AND CONTROL 8 (4), 338–353. doi: 10.2307/2272014

[B70] Zhang X. Liu X. Qi M. Liu Y. Kuai J. (2013). Alfalfa seeding root characteristics under complex saline-alkali stress. Chin. J. Eco-Agriculture 21 (3), 340–346. doi: 10.3724/SP.J.1011.2013.00340

